# Nitrogen and carbon source balance determines longevity, independently of fermentative or respiratory metabolism in the yeast *Saccharomyces cerevisiae*

**DOI:** 10.18632/oncotarget.8656

**Published:** 2016-04-08

**Authors:** Júlia Santos, Fernanda Leitão-Correia, Maria João Sousa, Cecília Leão

**Affiliations:** ^1^ Life and Health Sciences Research Institute (ICVS), School of Health Sciences, University of Minho, Braga, Portugal; ^2^ ICVS/3B's - PT Government Associate Laboratory, Braga/Guimarães, Portugal; ^3^ Molecular and Environmental Biology Centre (CBMA), Department of Biology, University of Minho, Braga, Portugal

**Keywords:** nitrogen source, prototrophic yeast, chronological life span, aging, Saccharomyces cerevisiae, Gerotarget

## Abstract

Dietary regimens have proven to delay aging and age-associated diseases in several eukaryotic model organisms but the input of nutritional balance to longevity regulation is still poorly understood. Here, we present data on the role of single carbon and nitrogen sources and their interplay in yeast longevity. Data demonstrate that ammonium, a rich nitrogen source, decreases chronological life span (CLS) of the prototrophic *Saccharomyces cerevisiae* strain PYCC 4072 in a concentration-dependent manner and, accordingly, that CLS can be extended through ammonium restriction, even in conditions of initial glucose abundance. We further show that CLS extension depends on initial ammonium and glucose concentrations in the growth medium, as long as other nutrients are not limiting. Glutamine, another rich nitrogen source, induced CLS shortening similarly to ammonium, but this effect was not observed with the poor nitrogen source urea. Ammonium decreased yeast CLS independently of the metabolic process activated during aging, either respiration or fermentation, and induced replication stress inhibiting a proper cell cycle arrest in G0/G1 phase. The present results shade new light on the nutritional equilibrium as a key factor on cell longevity and may contribute for the definition of interventions to promote life span and healthy aging.

## INTRODUCTION

Longevity regulation in yeast and in higher eukaryotes involves several regulatory mechanisms from nutrient-signaling pathways and autophagy to metabolic shifts in energy-generating processes [1-7]. Several of the major conserved pro-aging pathways have been extensively studied in yeast, with multiple studies in this eukaryotic model unraveling the relation between longevity, nutrients and metabolic shifts that regulate survival [8-11]. Caloric restriction (CR), reduction of caloric intake without compromising other nutrients, is a commonly used intervention known to extend life span in eukaryotic-aging models from yeast to mammals [12, 13]. CR longevity regulation is mediated through mechanisms overlapping with the major nutrient-signaling pathways [14-16]. Recently, an association of mitochondrial respiration with glucose aging signaling has also been described showing that the chronological life span (CLS) of respiratory-deficient strains with a respiratory capacity above the critical threshold during growth is extended by CR similarly to wild-type strains [8]. According to this study, calorie restricted cells presented a 30% increase in respiratory rate during growth in comparison to non-CR cells, but drastically reduced their respiratory capacity in stationary phase. This study presented evidence that the extended CLS encountered in CR cells can be achieved in non-CR cells by increasing respiratory capacity during growth only if accompanied by enhancement of cell stress resistance mechanisms that promote survival in stationary phase such as accumulation and mobilization of nutrient storage. CR has been linked to respiratory capacity in longevity regulation in yeast, by being able to promote the shift from fermentation to respiration and hence extend CLS [17]. Others have also reported metabolic changes in the Target Of Rapamycin- ortholog of the mammalian S6 kinase (TOR-SCH9) signaling inhibition that lead to acetic acid catabolism and consequently to trehalose accumulation and longevity promotion [18].

Ammonium, like other nutrients, has been implicated in dietary balance, a new concept that has only just emerged in longevity regulation [19]. This concept postulates that a balance between several nutrients, rather than glucose alone, regulates life and health span in a variety of models, such as yeast, flies and rodents [11, 20-22]. The Protein: Carbohydrate ratio (P:C) is a novel concept that emerged from several dietary studies, mainly in flies and rodents, that shows that the interaction between these two macronutrients can dictate longevity regulation [23, 24]. These studies postulate that higher P:C ratios lead to shortening of life span mainly through mTOR activation while lower P:C ratios increase longevity extension in the different models [22]. In a *Drosophila melanogaster* study, it was shown that while CR had no effect on life span extension, a 1P:16C ratio maximized this extension [25]. In yeast, although a specific P:C ratio that maximizes life span has not yet been established, several studies report the interaction between macronutrients such as glucose, Yeast Nitrogen Base (YNB), amino acids or ammonium as having a major impact on longevity regulation [11, 26-28]. As in yeast, studies in primates point to a dietary balance between protein and carbohydrate sources intimately connected to life and health span [29-31]. Ammonium has been implicated in CLS modulation in yeast, either in standard medium culturing conditions or under extreme CR in water, mainly in amino acid restricted cells, through the regulation of nutrient-signaling pathways such as TOR, SCH9 and Protein Kinase A (PKA) [10]. A link between specific amino acid deprivation and ammonium effects during aging was also reported, with these effects being mediated by Tor1p activation under leucine or histidine deprivation and by Ras2p activation under lysine deprivation [27].

Opposite to CR influence in CLS extension, growth signaling by increasing glucose from the standard 2% to 10% prevented an efficient stationary phase G0/G1 arrest, leading to DNA and replication stress, accompanied by increased levels of intracellular superoxide anion and decreased levels of H_2_O_2_ that culminated in CLS shortening [32, 33]. Likewise, the presence of ammonium in yeast culture medium, prevented an efficient G0/G1 arrest, showing that, like glucose, ammonium can induce replicative stress [28].

Here we present data on the role of single carbon and nitrogen sources and their balance on the longevity of the yeast prototrophic strain *S. cerevisiae* PYCC 4072, demonstrating that (i) ammonium decreases yeast CLS independently of the metabolic process active during aging, (ii) glutamine also induced CLS shortening, but this effect was not observed with the poor nitrogen source urea and (iii) CLS extension depended on a balance between ammonium and glucose, which also determined replication stress during CLS shortening.

## RESULTS

### Ammonium effects during aging and their dependence on glucose concentration

**Figure 1:**
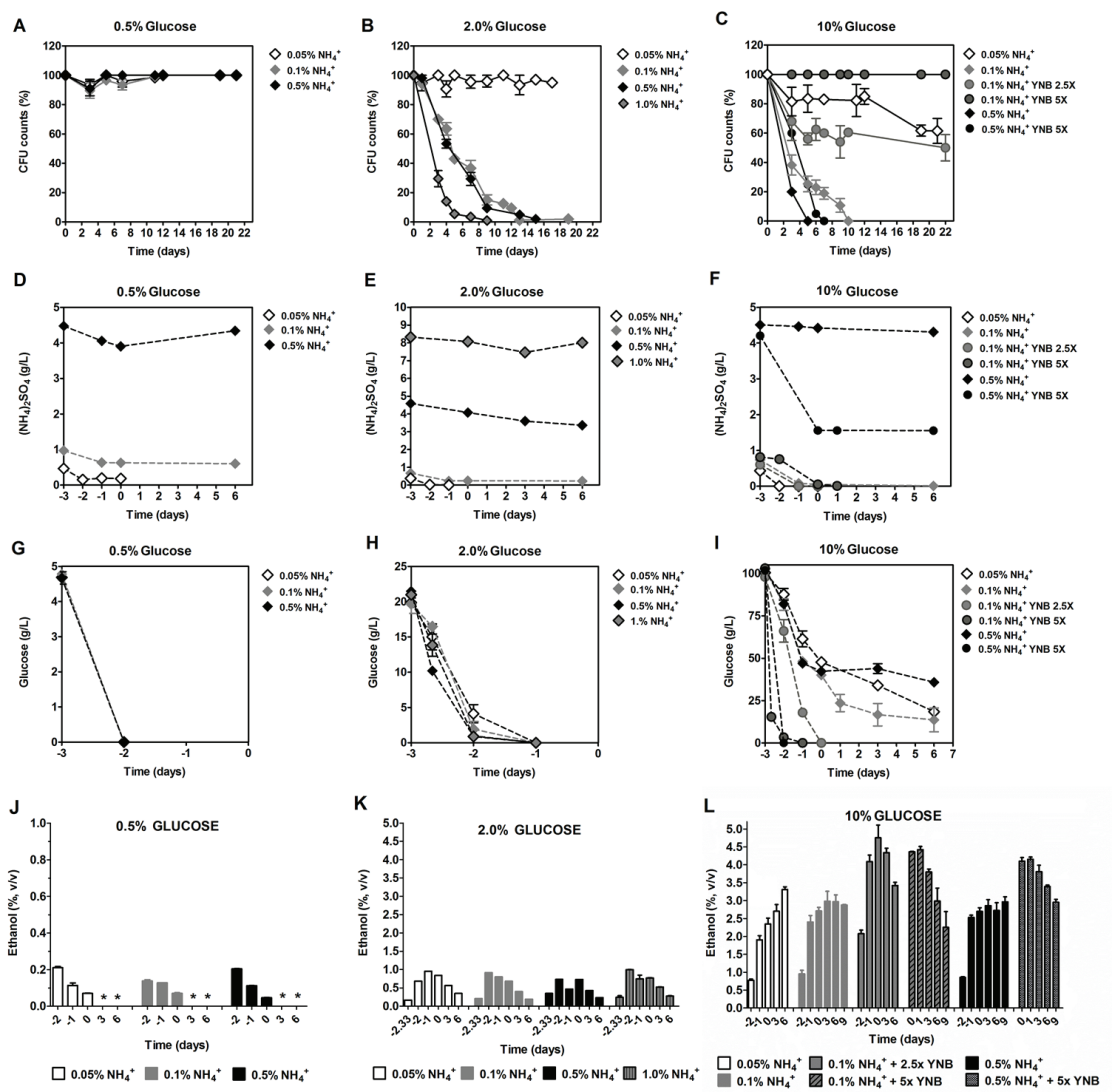
Survival **A., B.** and **C.**, (NH_4_)_2_SO_4_ consumption **D.**, **E.** and **F.**, glucose consumption **G.**, **H.** and **I.** and ethanol production/consumption **J.**, **K.** and **L.** of *S. cerevisiae* PYCC 4072 cells cultured in SD medium buffered to pH 3.4 with: 0.5% glucose (A, D, H and J); 2% glucose (B, E, I and K) and 10% glucose (C, F, I and L) and supplemented with different concentrations of (NH_4_)_2_SO_4_, (0.05%, 0.1%, 0.5% and 1.0%, NH_4_^+^) and Yeast Nitrogen Base (2.5x and 5x, YNB). Day −3 represents the day of culture inoculation and day zero represents the beginning of aging experiments. In all the cultures, starting cell density was about 3.8 × 10^7^ cells/ml. Values are means ± SEM (*n* = 3). (B) *P* < 0.001 (1.0% NH_4_^+^ *vs* 0.5% NH_4_^+^).

As mentioned above, ammonium has been described as an extrinsic factor that decreases CLS of auxotrophic strains and ammonium toxicity linked to essential amino acid limitation [10, 27, 28]. We now aimed to elucidate the role of ammonium on the CLS of yeast cells without amino acid restriction, and therefore a prototrophic yeast strain, not requiring amino acid supplementation, was used. We started by evaluating the CLS of the *S. cerevisiae* strain PYCC 4072 in medium containing 2% glucose and different concentrations of ammonium. As presented in Figure 1B, while for 0.05% ammonium sulphate (from now on, referred only as ammonium) no loss of cell viability was detected, concentrations above 0.1% led to a progressive CLS shortening, with almost no cell survival at day 9 for the highest ammonium concentration tested (1.0%).

We followed by questioning if extension of yeast CLS induced by ammonium restriction depends on the glucose concentration in the medium. Yeast CLS was therefore evaluated in the presence of low and high glucose concentrations in the medium, in combination with different ammonium concentrations (0.05%, 0.1% or 0.5%). Data showed that with 0.5% glucose, a condition commonly used in yeast as a caloric restriction condition [17], none of the three concentrations of ammonium used was able to induce CLS - shortening (Figure 1A). On the other hand, increasing glucose concentration to 10% increased the toxic effects of 0.1% and 0.5% ammonium, inducing shortening of the yeast CLS (Figure 1C). However, at 0.05% ammonium, a reversion of the negative effect associated with 10% glucose on cell survival was observed.

Next, we tested if differences in ammonium and glucose consumption during growth on the different media used could be conditioning the cellular environment during aging and, as a consequence, their CLS. We measured ammonium and glucose concentration in the medium from the beginning of growth (day −3) until exhaustion or until day 6 of CLS. Results show that, before day 0, ammonium was only fully consumed when the initial concentration was 0.05%, in the presence of 2% or 10% glucose (Figure 1E and 1F). For the other ammonium, concentrations (0.1% and 0.5%), ammonium was still present in medium at day 0, being totally consumed after day 0 for 10% glucose with 0.1% ammonium (Figure 1D-1F).

As for glucose consumption, in medium with 0.5% or 2% glucose, the sugar was totally depleted at day −2 (Figure 1G and 1H), independently of the ammonium concentration, although when the ammonium concentration was decreased down to 0.05% in 2% glucose medium there was a slight delay in glucose consumption (Figure 1H). In media with 10% glucose and 0.1% or 0.5% ammonium, glucose was not totally consumed which correlates with the rapid loss of cell viability in these conditions (Figure 1I). However, in the presence of 0.05% ammonium, despite glucose not being fully exhausted from the medium, a significant CLS shortening was not observed, with a cell survival of 70% at day 22 (Figure 1I). These results showed that in conditions where caloric restriction was not applied (2% and 10% glucose) maximum CLS extension was only attained when ammonium was totally consumed before aging. On the other hand, under caloric restriction (0.5% glucose), ammonium was still present, but did not have a negative impact on aging (Figure 1D).

**Figure 2:**
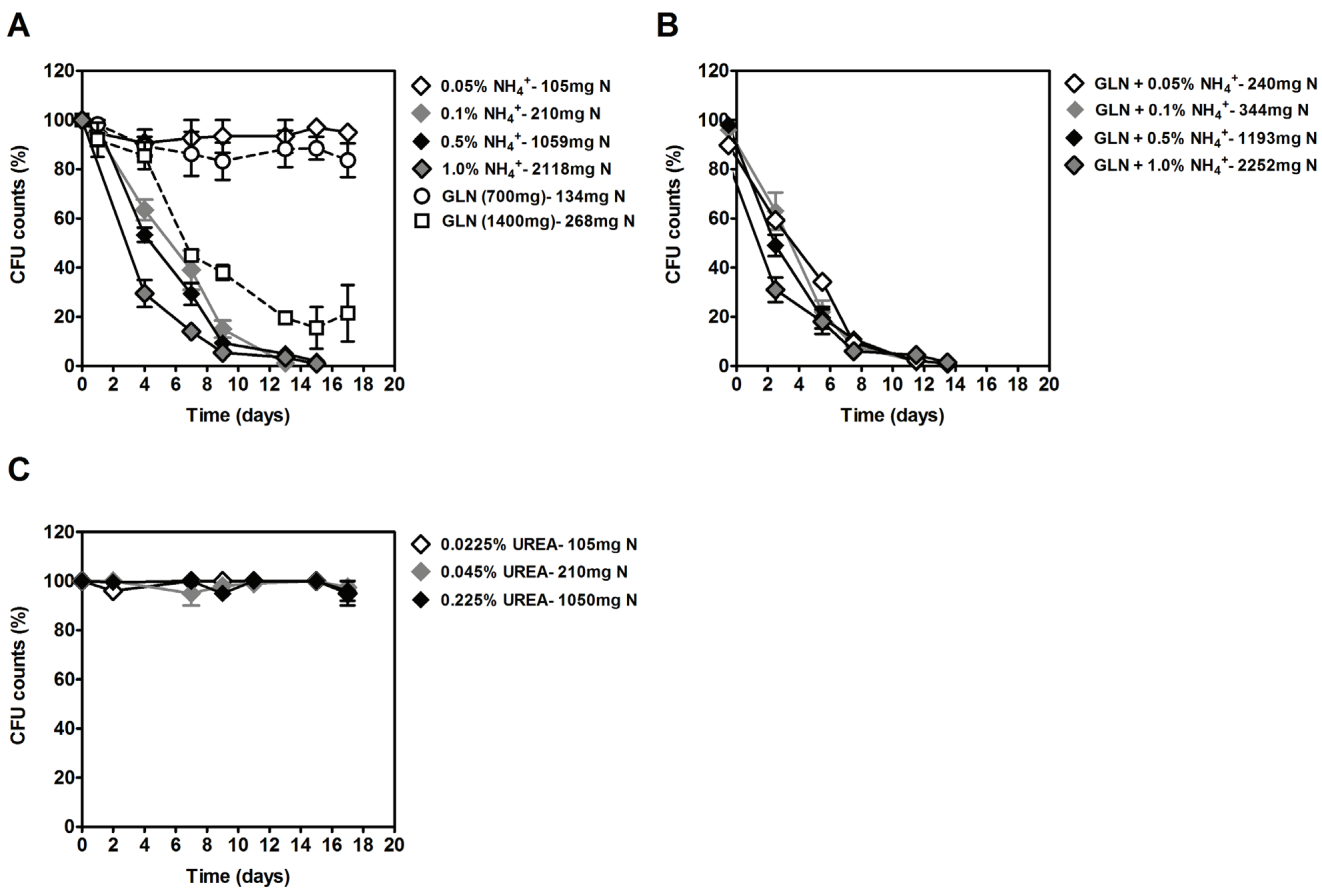
Survival of *S. cerevisiae* PYCC 4072 cells cultured in SD medium buffered to pH 3.4 with 2% glucose and supplemented with: different concentrations of (NH_4_)_2_SO_4_, (0.05%, 0.1%, 0.5% and 1.0%, NH_4_^+^) or glutamine (700 and 1400 mg/L) **A.** glutamine (700 mg/L) plus different concentrations of (NH_4_)_2_SO_4_ (0.05%, 0.1%, 0.5% and 1%; GLN + NH_4_^+^) **B.** different concentrations of urea (0.0225%, 0.045% or 0.225%, UREA) **C.** In all figures (A, B and C) total Nitrogen (N) concentration for each condition is indicated. In all the cultures, starting cell density was about 3.8 × 10^7^ cells/ml. Values are means ± SEM (*n* = 3).

We also investigated the effects of ammonium on yeast CLS in media with 2% glucose without or with other nitrogen sources (glutamine and urea) at several concentrations that result in equivalent total nitrogen (N) amounts in the medium. For the lowest ammonium and glutamine concentrations (105 and 134 mg/L N, respectively), no significant decrease on cell viability was observed (Figure 2A). However, in medium with glutamine as the only nitrogen source, doubling its concentration from 700 mg/L to 1400 mg/L (268 mg/L N) induced a rapid CLS shortening. We also tested the combined effects of ammonium plus glutamine (Figure 2B). For the medium with the lowest glutamine and ammonium concentrations (700 mg/L glutamine plus 0.05% (NH_4_)_2_SO_4_, 240 mg/l total N), a rapid loss of cell viability was observed. For higher ammonium concentrations, CLS shortening was even slightly higher.

The results obtained with urea were quite different (Figure 2C). For all the concentrations of urea used, even the highest 0.225% (1050 mg/L N), no loss of cell viability was observed. These results show that the CLS shortening induced by ammonium and glutamine is not observed with the poor nitrogen source urea.

### The maximum CLS extension could only be attained when nutrients of the YNB medium are not limiting ammonium or glucose consumption

As described above, in media supplemented with 10% glucose and with the higher ammonium concentrations (0.1% and 0.5%), where loss of cell survival occurred, both glucose and ammonium were not fully depleted (Figure 1C, 1F and 1I), suggesting that, in these conditions, other components could be acting as limiting nutrients. To assess this possibility, we increased YNB concentration by 2.5 and 5 fold. The results showed that with 0.1% ammonium and 10% glucose, increasing YNB either 2.5x or 5x, completely reverted the loss of cell viability, accompanied by a faster glucose consumption (Figure 1C and 1I). Regarding ammonium consumption, it was depleted at day 0 both in 2.5x and 5x YNB media (Figure 1F). However, in medium supplemented with 0.5% ammonium and 10% glucose, increasing YNB 5x did not have any beneficial effect on yeast CLS. Instead, increasing YNB boosted a rapid glucose depletion from the medium, after which ammonium consumption stopped, with approximately 0.15% remaining in the medium (Figure 1C, 1F and 1I). In summary, the results indicate that the maximum CLS extension could only be attained when YNB nutrients are not limiting glucose and ammonium consumption, and therefore ammonium or glucose were totally consumed before aging.

### Respiratory or fermentative metabolism do not relate with the yeast cell longevity

To further evaluate if a metabolic shift between fermentation and respiration may take place under the different glucose and ammonium concentrations, fermentation occurrence was evaluated by measuring ethanol production in the experiments above (Figure 1J, 1K and 1L). For all conditions tested, ethanol was always produced with a stoichiometry that was consistent with a predominant glucose fermentative metabolism. In fact, considering a theoretical yield of 0.9, for example 2% glucose would produce 1.06 (v/v) ethanol which is very close to the experimental values (Figure 1K). Consumption of the produced ethanol could be observed after sugar exhaustion, indicating a shift to a respiratory metabolism. For the lower glucose concentration (0.5%), ethanol was completely consumed between day 0 and day 3. For 2% glucose, although a similar pattern was observed, ethanol was still present in the medium at day 6, mainly due to its higher initial production (Figure 1J and 1K). For 10% of glucose in 1x YNB, the limitation of sugar consumption was also reflected on ethanol production which was still increasing after day 0 during CLS. In turn, supplementation with 2.5x or 5x YNB allowed a faster ethanol production, reaching the maximum ethanol concentration at day 0, after which its consumption started (Figure 1L).

The analysis of these results with those from the survival curves appear to indicate that CLS extension could be observed both for cells fermenting glucose (e.g. Figure 1C and 1L, condition 10% glucose and 0.05% ammonium) or oxidizing the produced ethanol (e.g., Figure 1A and 1J) along aging experiments, suggesting no apparent relation between oxidative or fermentative metabolism and cell longevity. To further sustain this hypothesis, we evaluated the respiration capacity (RC) of cells from aging experiments under conditions representative of the two metabolic states (Table 1). As expected, for all the glucose and ammonium concentrations tested, the RC of exponential cells was inversely correlated with the initial glucose concentration in the medium, increasing as the sugar was consumed. After glucose exhaustion, the consumption of the produced ethanol started (post-diauxic phase), which was accompanied by further increase in the cell RC.

**Table 1:** Respiratory capacity (RC - O_2_ consumed mmol/min/mg) of yeast cells at different time points* (T_−2.33_; T_−2_; T_−1_; T_0_) of the growth curve in the different conditions tested.

Culture condition	T_−2.33_	T_−2_	T_−1_	T_0_
**0.5% Glu + 0.05% NH_4_^+^**	539	749	959	314
**0.5% Glu + 0.10% NH_4_^+^**	521	1009	1054	327
**2.0% Glu + 0.05% NH_4_^+^**	409	372	361	472
**2.0% Glu + 0.10% NH_4_^+^**	408	366	369	641
**10% Glu + 0.05% NH_4_^+^**	215	214	225	198
**10% Glu + 0.10% NH_4_^+^**	218	225	136	109
**10% Glu + 0.10% NH_4_^+^ 2.5X YNB**	278	264	372	345

In agreement with the results of ethanol production/consumption, the analyses of the RC of cells at T0 and those of the corresponding survival curves (Figure 1B) also show that CLS shortening could occur in cells displaying either the highest (Table 1, line 4 ) or the lowest (Table 1, line 6) RC values. Likewise, CLS extension could occur both in cells with higher (Table 1, lines 2, 3 and 7; Figure 1A, 1B and 1C) or lower (Table 1, line 5; Figure 1B) RC values at T0. These data indicate that respiratory or fermentative metabolism do not relate with the yeast cell longevity.

### The negative effects of ammonium observed during yeast aging are associated with replication stress

**Figure 3:**
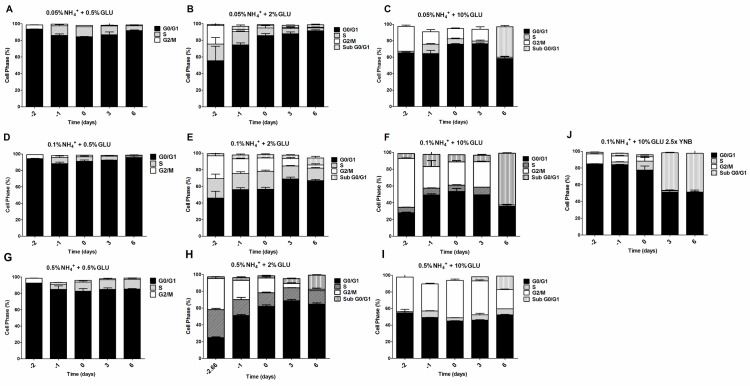
Cell cycle analysis of *S. cerevisiae* PYCC 4072 cells cultured in SD media buffered to pH 3.4 with 0.5% glucose **A.**, **D.** and **G.** 2% glucose **B.**, **E.** and **H.** and 10% glucose **C.**, **F.**, **I.** and **J.** and supplemented with different concentrations of (NH_4_)_2_SO_4_ (NH_4_^+^): 0.05% (A, B and C); 0.1% (D, E, F and J) and 0.5% (G, H, I); and Yeast Nitrogen Base (2.5x, YNB) (J). Day −3 represents the day of culture inoculation and day zero represents the beginning of aging experiments. In all the cultures, starting cell density was about 3.8 × 10^7^ cells/ml. Values are means ± SEM (*n* = 3).

To further evaluate if the nitrogen effects on prototrophic yeast longevity and their interplay with glucose metabolism are linked to replicative stress, we monitored cell cycle progression by flow cytometry along the aging experiments under the different conditions tested. In media with 0.5% glucose and 0.05%, 0.1% or 0.5% ammonium supplementation, a proper cell cycle arrest in G0/G1 phase was observed that coincided with glucose exhaustion, with more than 90% of the cells arrested in this phase (Figure 3A, 3D and 3G). For 2% glucose media, supplementation with increasing ammonium concentrations seemed to decrease the percentage of cells with a proper cell cycle arrest in G0/G1 phase (Figure 3B, 3E and 3H). In fact, in media with 2% glucose and 0.05% ammonium, cells entered a proper cell cycle arrest with more than 90% of cells in G0/G1 phase, while only about 60% of cells reached a proper cell cycle arrest in media with 0.1% or 0.5% ammonium. In media with 10% glucose and 0.05% ammonium, about 70% of the population reached G0/G1 phase arrest (Figure 3C). On the contrary, in media with 10% glucose and 0.1% or 0.5% ammonium, cells could not enter a proper cell cycle arrest (Figure 3F and 3I). However, supplementation of media with 10% glucose and 0.1% ammonium with 2.5x YNB led to cell cycle arrest with almost 85% of cells arresting in G0/G1 phase, in agreement with the rapid exhaustion of glucose in this condition (Figures 1C and 3J).

The analysis of data from Figures 1 and 3 also showed that CLS extension was observed when more than about 80% of cell population enters the G0/G1 phase, while for the conditions where ammonium induced CLS shortening a proper cell cycle arrest was not achieved (Figure 3E, 3F, 3H and 3I). Together, these results indicate that the negative effects of ammonium observed during the prototrophic yeast aging are associated with replication stress induction.

## DISCUSSION

In the present work, we started by uncovering the effects of ammonium on the CLS of *S. cerevisiae* PYCC 4072. The first note to highlight is that ammonium was capable of decreasing CLS of this prototrophic strain as previously observed in auxotrophic strains [28]. The effects were mainly reliant on the ammonium and glucose concentration in the medium. Taken together, data suggest that CLS shortening could only be observed for high concentrations of ammonium and glucose in the aging cultures. Therefore, CLS extension could be attained by restriction of ammonium independently of imposing caloric restriction through lowering glucose concentration.

Another aim of the work was to explore the influence of other nitrogen sources on yeast CLS, namely: glutamine, a preferred nitrogen source like ammonium, and urea, a non-preferred nitrogen source. Similarly to ammonium, glutamine induced CLS shortening, indicating that the total N concentration, rather than the nature of the nitrogen source, is responsible for the decrease of yeast CLS when either of the two preferred nitrogen sources are present at high concentrations. Oppositely, such a CLS shortening was not observed when equivalent total N amounts of urea were used. The utilization of different nitrogen sources in yeast is controlled by fine regulation of specific pathways such as the Nitrogen Catabolic Repression (NCR) pathway, which allows the use of preferred nitrogen sources by repressing genes associated with the use of poorer nitrogen sources, and the TOR pathway [34]. Since signaling and assimilation pathways are differently activated by preferred or non-preferred nitrogen sources, our results suggest that these pathways could be involved in the regulation of CLS by nitrogen. Accordingly, previous results showed that ammonium-induced CLS shortening was associated with activation of both PKA and TOR pathways [10, 27].

Another interesting point is that the maximum CLS extension could only be attained when nutrients of the YNB medium were not limiting either nitrogen and/or carbon source consumption, and thereby allowing the total consumption of at least one of them before aging. As to the nature of the limiting nutrient in YNB, it is known that the availability of vitamin-derived enzyme cofactors, such as NADH/NADPH, thiamine pyrophosphate or biotin, is essential for metabolic processes [35, 36]. In this line, limitation of vitamins may be underlying the observed incomplete consumption of either nitrogen and/or carbon source and the consequent CLS shortening, but further studies are necessary to clarify this point.

Regarding the glucose metabolism under the different ammonium concentrations tested, in all experimental conditions a fermentative metabolism was present, at least during growth, as demonstrated by the production of ethanol. In *S. cerevisiae*, the beneficial outcomes of CR on life span extension have been linked to the derepression of aerobic catabolism, a phenomenon known as the Crabtree effect [37]. In our studies, although in CR conditions (0.5% glucose) a significant increase of CLS for all ammonium concentrations tested was observed as expected, this effect was independent of a respiratory metabolism previously described to be associated with CR conditions [17]. Similarly, for the higher glucose concentrations, there was no apparent relation between oxidative or fermentative metabolism and the cell longevity. Reinforcing this conclusion, the results obtained along aging experiments show that the yeast CLS extent is not strictly related with the respiratory capacity of the cells.

The effects on CLS of increasing glucose up to 10% have been described as associated with DNA replicative stress [33]. Our results further show that CLS extension is dependent on a proper cell cycle arrest that is obtained due to either glucose or ammonium exhaustion.

As a final remark, data herewith highlight the balance of nitrogen and carbon sources as major longevity regulators, in parallel to what has been described for the Protein: Carbohydrate ratio in higher eukaryotic models, further establishing yeast as an up to date aging model organism.

## MATERIALS AND METHODS

### Strains and growth conditions

*Saccharomyces cerevisiae* PYCC 4072 was used in this study. This strain was originally isolated from a sample of Fermivin, industrial wine yeast distributed by Rapidase, and was obtained from the Portuguese Yeast Culture Collection (PYCC), New University of Lisbon, Portugal. For the aging experiments, cells were cultured at 26¼C, 150 rpm, until stationary phase was reached, in defined minimal medium (SD medium) containing: 0.17% (1x), 0.425% (2.5x) or 0.85% (5x) yeast nitrogen base without amino acids and without ammonium sulphate (Difco, BD); 0.5%, 2% or 10% D-glucose; supplemented with ammonium sulphate (0.05%; 0.1%; 0.5% or 1.0%); glutamine (700 and 1400 mg/L) ); or urea (0.0225%; 0.045% or 0.225%) in different combinations. Citrate phosphate was used for buffering medium to pH 3.4 (28.2 mM Na_2_HPO_4_ and 35.9 mM citric acid). Cell cultures were inoculated with a starting Optical Density (O.D._640_) of 0.030. After 72 hours, cells were collected by centrifugation and resuspended at a cell density of 3.8 x10^7^ cells/ml in the same culture medium. This day was considered day zero of the CLS experiment and consequently inoculation day was considered day −3. For medium supplemented with urea stationary phase was reached only 10 days after inoculum (240 hours of growth), so CLS experiment was initiated only at this time point (day 0). The respective growth curves are presented in Supplementary data (Figure 1S). Cell viability was assessed by Colony Forming Units (CFU) at day 0 and in subsequent days by collecting culture aliquots that were subsequently spread on YEPD (2% glucose, 2% agar, 1% peptone and 0.5% yeast extract) agar plates for 2 days at 30¼C. CFU counts of day zero was considered to be 100% of survival of the aging experiment.

### Ammonium and glucose determination

To determine ammonium and glucose concentrations in the culture media, the cultures were sampled at the indicated time points and centrifuged for 5 min. The supernatant was frozen and kept at −20¼C until subsequent analysis. Ammonium and glucose were quantified using an ammonia assay kit (Sigma) and a glucose oxidase (GOD) assay (Roche Diagnostics GmbH), respectively and following the manufacturer's instructions.

### Cell cycle analysis

To measure DNA content, cells were stained with SYBR Green I as previously described [38] and staining was assessed by flow cytometry. Flow cytometry analysis of the experiments was performed in a BD™ LSR II flow cytometer and thirty thousand cells per sample were analyzed. Offline data was analyzed with the flow cytometry analysis software package FlowJo 7.6.1.

### HPLC quantification of ethanol

To determine ethanol concentrations in the culture media, the cultures were sampled and centrifuged for 5 minutes. The supernatant was frozen and kept at −20¼C until subsequent analysis. Ethanol quantification was assessed by high-performance liquid chromatography, using a refractive index detector IOTA2 from Gilson and a carbohydrate H^+^ column (SS-100, H^+^, 8μm, Hypersil), maintained at 54¼C. A solution of H_2_SO_4_ (0.0025 M) was used as the mobile phase at a flow rate of 0.7 ml/min.

### Assessment of respiratory capacity

For estimating the respiratory capacity (RC) of cells in the different conditions tested, cells were harvested, washed with H_2_O and resuspended in H_2_O (O.D._640nm_ = 40). A Clark electrode connected to an YSI 5300 monitor and to a recorder (Kipp & Zonen), was used. The electrode was immersed in a water chamber stirred with a magnet bar. 4.65 ml of deionized water and 0.2 ml of yeast suspension were added to the chamber, and a baseline was obtained. Subsequently 100 μl of glucose 1 M was added, and the oxygen consumption was followed in the recorder. The RC values were calculated from the slopes of the trace after the addition of glucose and normalized to the dry weight of the respective cell culture.

### Statistical analysis

Values presented in graphs represent means and standard deviations from three independent experiments (± SEM *n* = 3). Statistical analyses were performed by two-way ANOVA. *P* < 0.05 was considered statistically significant.

## SUPPLEMENTARY MATERIAL FIGURE


