# Supplementary material for: Nitrogen and carbon source balance determines longevity, independently of fermentative or respiratory metabolism in the yeast Saccharomyces cerevisiae
Source: Oncotarget. 2016 Apr 8;7(17):23033–42. doi: 10.18632/oncotarget.8656 (PMC5029608; doi:10.18632/oncotarget.8656)
Supplement: Supplementary file 1 [file oncotarget-07-23033-s001.pdf]

Nitrogen and carbon source balance determines longevity, independently of fermentative or respiratory metabolism in the yeast *Saccharomyces cerevisiae*

Supplementary Material

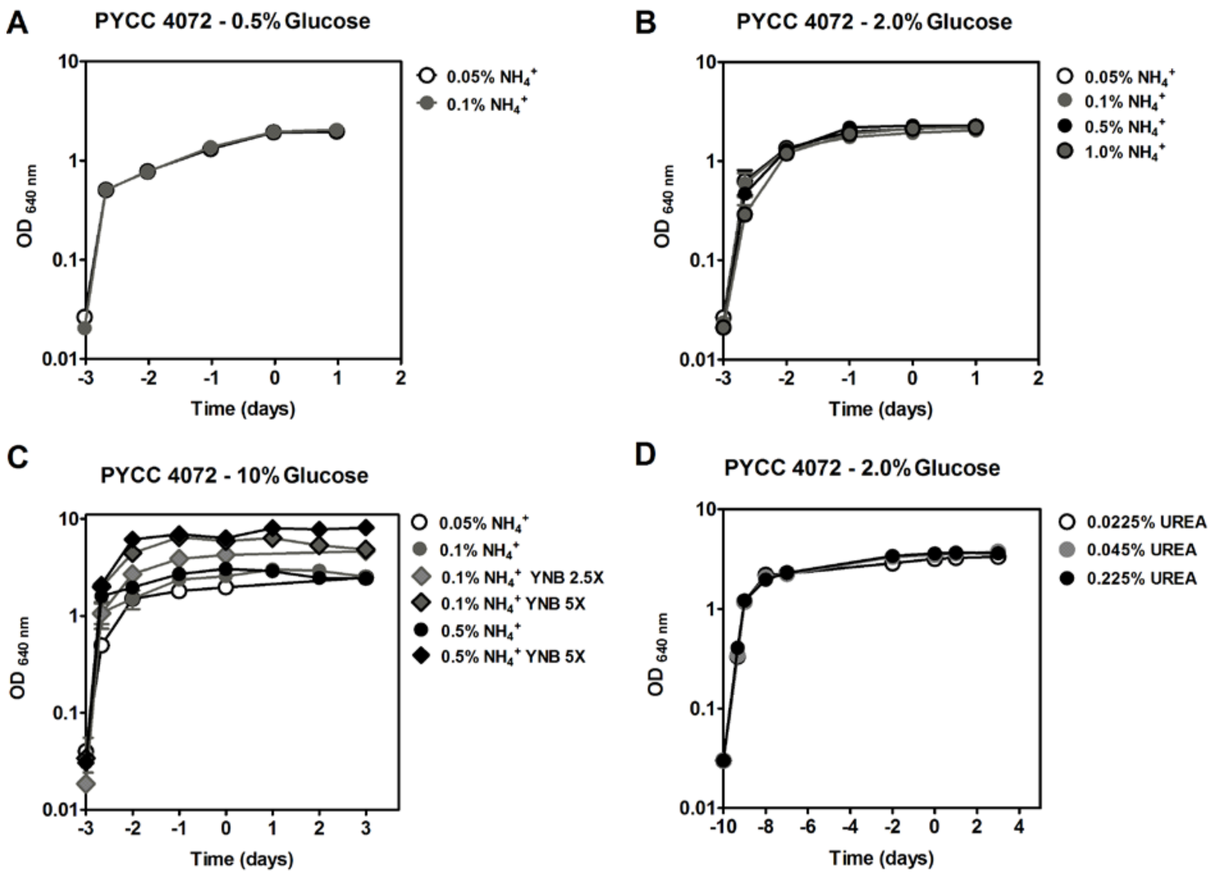

Figure S1. Growth curves of *S. cerevisiae* PYCC 4072. Cells were cultured in SD medium buffered to pH 3.4 with 0.5% glucose (A); 2% glucose **(B and D)** and 10% glucose **(C)** and supplemented with different concentrations of: $(NH_4)_2SO_4$, (0.05%, 0.1%, 0.5% and 1.0%, NH_4^+) **(A, B and C)**; Yeast Nitrogen Base (2.5x and 5x, YNB) **(C)** and urea (0.0225%, 0.045% and 0.225%) **(D)**.
